# 1823. Disproportionate Rates of COVID-19 Among Black Canadian Communities: Lessons from the First Year of the Pandemic

**DOI:** 10.1093/ofid/ofad500.1652

**Published:** 2023-11-27

**Authors:** Upton D Allen, Michelle Barton, Julia Upton, Annette Bailey, Aaron Campigotto, Mariana Abdulnoor, Jean-Philippe Julien, Jonathan Gubbay, Niranjan Kissoon, Alice Litosh, Peter Wong, Andrew Allen, Renee Bailey, Walter Byrne, Matthew Hwang, Chantal Phillips, Alicia Polack, Cheryl Prescod, Kimberly Thompson, Sylvanus Thompson, Nicole Wisener, Carl James

**Affiliations:** Hospital Sick Children, Toronto, Ontario, Canada; London health Sciences Centre, London, Ontario, Canada; Hospital for Sick Children, Toronto, Ontario, Canada; Toronto Metropolitan University, Toronto, Ontario, Canada; Hospital for Sick Children, Toronto, Ontario, Canada; Hospital for Sick Children, Toronto, Ontario, Canada; Hospital for Sick Children, Toronto, Ontario, Canada; Hospital for Sick Children, Toronto, Ontario, Canada; British Columbia Children's Hospital, Toronto, Ontario, Canada; Hospital for Sick Children, Toronto, Ontario, Canada; Hospital for Sick Children, Toronto, Ontario, Canada; Hospital for Sick Children, Toronto, Ontario, Canada; Hospital for Sick Children, Toronto, Ontario, Canada; Hospital for Sick Children, Toronto, Ontario, Canada; Hospital for Sick Children, Toronto, Ontario, Canada; Hospital for Sick Children, Toronto, Ontario, Canada; Hospital for Sick Children, Toronto, Ontario, Canada; Black Creek Community Health Centre, Toronto, Ontario, Canada; Hospital for Sick Children, Toronto, Ontario, Canada; Hospital for Sick Children, Toronto, Ontario, Canada; The Hospital for Sick Children, Thornhill, Ontario, Canada; York University, Toronto, Ontario, Canada

## Abstract

**Background:**

Black North American communities have been disproportionately affected by COVID-19. These data have been largely based on case counts, hospitalizations and mortality data. Serologic testing enables a more complete determination of infection burden by documenting infection in persons with symptomatic as well as asymptomatic infection. We used serologic testing to determine the extent to which SARS-CoV-2 had penetrated into the Black community. We examined risk factors associated with seropositivity, including the presence of medical comorbidities and the social determinants of health.

**Methods:**

We conducted a cross-sectional survey in a COVID-19 high-prevalence zone in Ontario along with 2 areas that have lower rates of COVID-19 cases. SARS-CoV-2 IgG antibodies were determined using the EUROIMMUN assay. The study samples were collected between August 15, 2020, and December 15, 2020 prior to the deployment of COVID-19 vaccines. Proportions were compared using Fishers Exact test or chi-square; potential risk factors were examined using a multiple logistic regression approach.

**Results:**

Among 387 evaluable subjects, the majority, 274 (70.8%) were enrolled from northwest Greater Toronto Area (GTA) and adjoining suburban areas of Peel, Ontario with a high proportion of Black residents. The seropositivity rates for the lower prevalence areas (Oakville and London, Ontario) were comparable (3.3% (2/60; 95% CI 0.4-11.5) and 3.9% (2/51; 95% CI 0.5-13.5), respectively). The seropositivity rate for the northwest GTA was 12.6% (26/206); RR 3.5, 95% CI 1.3-9.8). Persons under the age of 19 years had the highest seropositivity rate (10/50; 20.0%, 95% CI 10.3-33.7%). Front-line workers were greater than 3 times more likely to be seropositive compared with non-frontline workers (13.0 vs 3.2%; p=.01; RR 3.3 (95% CI 1.3 – 8.3). There was an interaction effect between race and location of residence as this relates to the relative risk of seropositivity.

**Conclusion:**

During the pre-vaccine phase of the COVID-19 pandemic, the seropositivity rate for SARS-CoV-2 within a COVID-19 high-prevalence area was 3-fold greater than lower prevalence areas of Ontario, Canada. The data help to define the burden of COVID-19 within a community with a high proportion of Black residents compared with other communities.

**Disclosures:**

**All Authors**: No reported disclosures

